# Terahertz wave stimulation improves visual recognition memory via the activation of excitatory neurons in the perirhinal cortex

**DOI:** 10.1186/s12964-026-03051-3

**Published:** 2026-07-06

**Authors:** Hui Wang, Zhiwei He, Lequan Song, Ji Dong, Junmiao Pan, Wenjing Cheng, Rui Wang, Yuqing Wang, Li Zhao, Ruiyun Peng

**Affiliations:** 1 Beijing Institute of Radiation Medicine, Beijing, 100850 China; 2 Bioinformatics Center of AMMS, Beijing, 100850 China

**Keywords:** Terahertz, Recognition memory, Excitatory neurons, Glutamate receptors, Synaptic transmission

## Abstract

Terahertz (THz) waves, with frequencies between those of microwaves and infrared light, can excite nonlinear resonance and affect cellular function. However, the effects and underlying mechanism of terahertz radiation on neurons and rodent behaviour are still unclear. Here, we constructed THz radiation exposure models in the perirhinal cortex (PRH) of male mice. First, we measured the power and depth of penetration of the 0.152 THz photons cranially above the PRH. We demonstrated that the visual recognition memory of mice was improved after THz wave irradiation, which was similar to the effect of light activation of glutamatergic neurons in the PRH. In addition, THz wave exposure increased the number of c-Fos^+^ neurons, synchronous electrical activity, synaptic plasticity and glutamatergic neuronal excitability in the PRH. Moreover, the expression of molecules involved in glutamate transmission, such as VAMP2, EAAT, CaMKII and PSD95, increased along with enhanced NMDAR and AMPAR activity. Our findings revealed that THz waves could improve recognition memory via the activation of excitatory neurons and accelerated glutamate metabolism in the PRH, suggesting that THz photon stimulation might be developed as a noninvasive neuromodulation technique to promote cognitive function.

**Supplementary Information:**

The online version contains supplementary material available at https://doi.org/10.1186/s12964-026-03051-3.

## Introduction

THz waves are electromagnetic waves with a frequency range of 0.1–10 THz and a wavelength of 30–3000 μm. With the progress in THz source-connected materials and optical elements, the traditional “THz gap” has been filled [1]. THz wave stimulation is considered to affect the nervous system because of its abundant neural electrical activity. Researchers have shown that THz waves can increase excitatory synaptic transmission and neuronal firing activities and promote the dynamic growth of the neuronal cytosol and protrusions [2, 3]. In addition, in the higher midinfrared band, the neuromodulatory roles of electromagnetic waves have attracted the attention of researchers. Increased activities of cortical neurons and acceleration of associative learning in zebrafish were found in the 30–55 THz band [4–6]. However, infrared neural stimulation is believed to lead to a greater temperature increase (> 1 °C) [6]. Therefore, identifying neuromodulation methods that effectively regulate neural activity while minimizing adverse effects and thus maximizing the nonthermal effects of low-frequency terahertz waves is crucial.

Visual recognition memory is the ability to discriminate whether a visual stimulus is novel or familiar, which is essential for daily life. Recognition memory can be assessed with the novel object preference (NOP) test, the novel location preference (NLP) test and the object-in-place (OiP) test [7]. The PRH is a juxtallocortex that transitions between the hippocampal formation and the neocortex of the temporal lobe and is essential for recognition memory [8]. The mechanisms responsible for recognition memory in the PRH are connected with synaptic plasticity by electrophysiological recordings from slices of the PRH and with transmitter receptors or downstream cascades of synaptic plasticity [9]. Numerous previous studies have shown that optical manipulation of activity in neural circuits with light-sensitive rhodopsin may aid in understanding memory and treating cognitive deficits. However, the optogenetics technique has shown little potential for applications in healthy humans because exogenous genes must be introduced into the brain. Therefore, it is critical to develop a new brain stimulation scheme without introducing exogenous genes or significant thermal effects.

One of our previous studies showed that 0.16 THz with an output power of 50 mW wave radiation for 60 min induced an increase in glutamate in primary cortical neurons, indicating that the neurons were in excitatory states [10]. Moreover, 0.152 THz wave exposure for 60 min significantly increased the activity of glutamatergic neurons and improved spatial working memory [11]. In contrast to the research involving high-frequency terahertz and mid-infrared spectra where thermal effects are prominent, the biological responses to low-frequency terahertz waves are predominantly driven by non-thermal effects [12]. Current studies on low-frequency THz waves have focused mainly on the cellular level and have lacked systematic studies on multiple levels, especially the animal level [2, 13–15]. Therefore, in this study, we discuss the effects of THz waves with a frequency of 0.152 THz on cortical neurons in the PRH area. Here, we present a new physical stimulation method for improving visual recognition memory at the brain slice and animal levels. We demonstrated that 0.152 THz waves could improve visual recognition memory by activating excitatory neurons without differences in optogenetic enhancement. We also found that synaptic plasticity, notably long-term potentiation (LTP) in vitro, significantly increased after THz stimulation. The underlying mechanism might be related to glutamate-mediated synaptic transmission.

## Materials and methods

### Animals

All animal studies and experimental procedures were approved by the Animal Care and Use Committee (IACUC-DWZX-2024-574). C57BL/6 N male mice (6–8 weeks) were raised in a specific pathogen-free (SPF) environment in a laboratory animal facility at temperatures ranging from 21 °C to 23 °C and humidity levels between 40% and 60% on a 12-h light/dark cycle. Standard rodent food and water were freely available to the mice. All experiments were performed exclusively during the daylight period of the light/dark cycle, strictly adhering to institutional guidelines.

The mice or brain slices were divided into two groups: a control (C) group and a THz wave exposure (T) group. The T group was exposed to 0.152 THz radiation for 60 min, whereas the C group was not exposed to THz waves.

### Brain slice preparation

The mice were anaesthetized with pentobarbital sodium. The brains were quickly excised and perfused with 20 ml of oxygenated ice-cold modified artificial cerebrospinal fluid (ACSF) (composition: 10 mM NaCl, 2.5 mM KCl, 1.25 mM NaH_2_PO_4_, 25 mM NaHCO_3_, 0.5 mM CaCl_2_, 7 mM MgCl_2_, 195 mM sucrose, 10 mM glucose, and 2 mM sodium pyruvate; osmolarity 300–305 mOsm/kg, pH 7.35–7.45 adjusted with NaOH, 95% O_2_/5% CO_2_). Fresh brain slices (300 μm) containing the PRH cortex (bregma: -1.34 mm~ -2.18 mm) were cut using a vibrating microtome (Campden, UK). The brain slices were allowed to recover for 30 min in ACSF at 32 °C and then incubated at room temperature for at least 1 h with the oxygenated standard ACSF (composition: 119 mM NaCl, 2.5 mM KCl, 1.25 mM NaH_2_PO_4_, 24 mM NaHCO_3_, 2 mM CaCl_2_·2H_2_O, 2 mM MgSO_4_·7H_2_O, and 12.5 mM glucose; osmolarity 300–305 mOsm/kg, pH 7.35–7.45 adjusted with NaOH). All the chemicals used for slice preparation were purchased from Sigma.

### THz wave exposure

A backwards wave oscillator (BWO)-based THz source with a frequency range of 96–180 GHz (QS2-180, Microtech Instruments, USA) was used to generate THz waves. In addition, for the BWO-based THz wave source, each frequency corresponds to a specific output power [10].

**Fig. 1:**
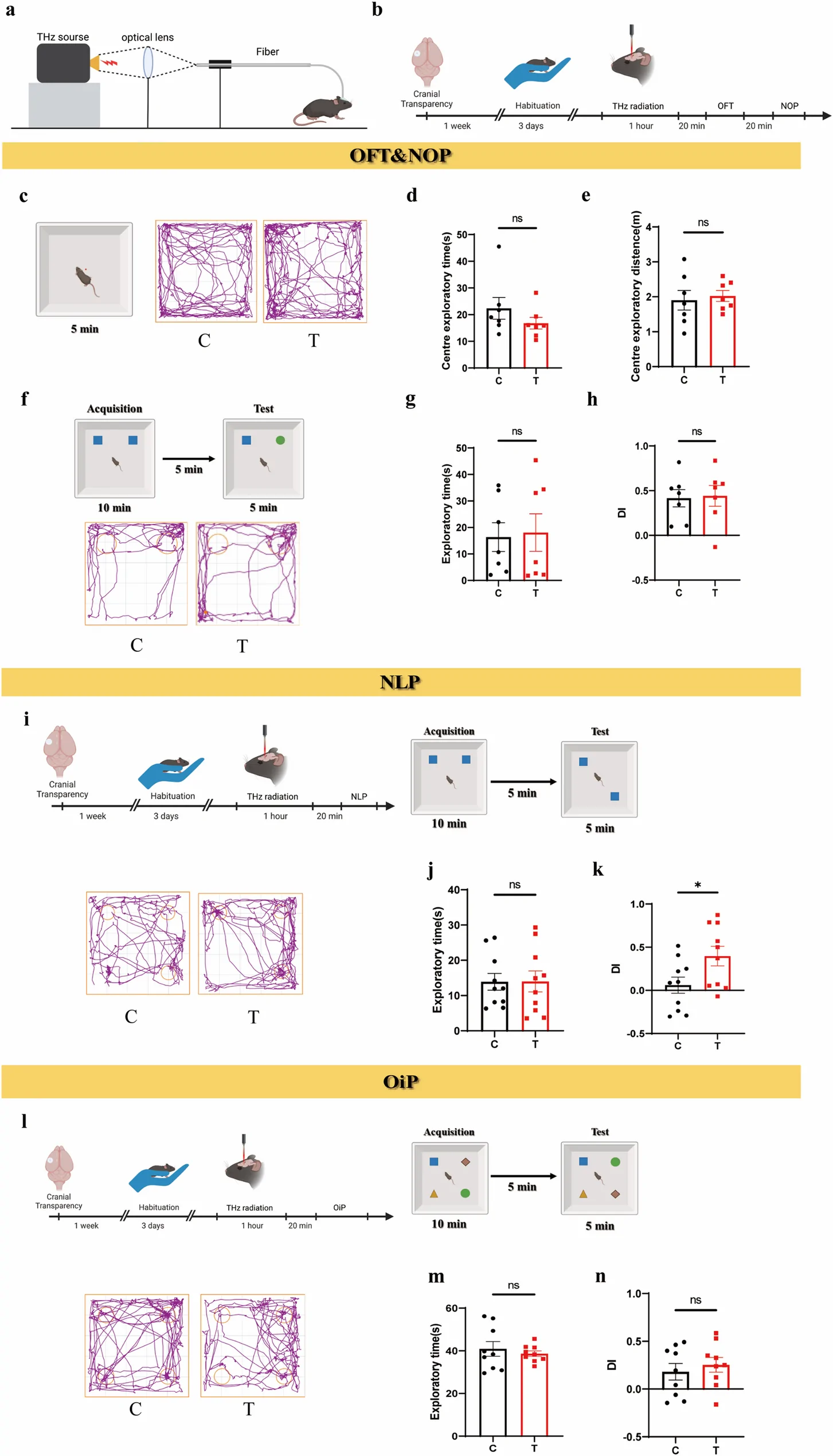
Improved location recognition memory in mice after THz wave exposure. **a** Schematic diagram of a mouse irradiated with THz waves. **b** Flowchart of the cranial transparency surgery and subsequent OFT and NOP tests. **c** Schematic diagram of the OFT and traces of mouse movement between the C and T groups. **d**-**e** Anxiety-like behavioural data analysis during the OFT (*n* = 7 mice). **f** Schematic diagram of the NOP test and traces of mouse movement between groups. **g**-**h** Changes in object recognition memory in mice irradiated by THz waves (*n* = 7 mice). **i** Flowchart and schematic diagram of the NLP test and traces of mouse movement between groups. **j**-**k** Changes in location recognition memory in mice irradiated by THz waves (*n* = 10 mice). **l** Flowchart and schematic diagram of the OiP test and traces of mouse movement between groups. **m**-**n** Changes in object-in-place recognition memory in mice irradiated by THz waves (*n* = 9 mice). All the data are presented as the means ± SEMs. Significance was assessed using an unpaired *t* test. *p < 0.05, ns (not significant)

For the mouse model, Krazy Glue^®^ (Elmer’s Products, USA) was used to obtain a transparent skull above the PRH cortex (Fig. S1a) [16]. The mice were anaesthetized and fixed in a custom-made device (Fig. 1a, Fig. S1b). Conductive fibres made of polytetrafluoroethylene were placed at the focus of a convex lens (Fig. S1c). Therefore, terahertz waves could pass through the convex lens and conductive fibre and finally act on the PRH. For the loss of the optical path, the power output was measured as 7.15 mW by a Vector H410 power metre (Scientech, USA; Fig. 1a).

For in vivo experiments, skull transparency was performed one week prior to the initiation of the studies. For mice assigned to optogenetic and chemogenetic groups, viral injections and skull transparency was conducted three weeks in advance to ensure adequate viral expression and surgical recovery. Following recovery, all mice underwent a three-day habituation period, after which they were subjected to THz wave irradiation and behavioural assessments. Detailed temporal parameters for these procedures are provided in the Results section. According to our previous study, the temperature increase at the skull surface during 60 min of exposure was 0.57 ± 0.26 °C [11].

For in vitro experiments, after brain slice preparation, the slices were incubated in oxygen-saturated ACSF for 30 min to allow for physiological recovery. Subsequently, the slices were subjected to 0.152 THz wave irradiation for 60 min. Following the exposure, electrophysiological recordings, calcium imaging, immunofluorescence, and Western Blot analyses were performed immediately (Fig. S2c). The THz wave exposure conditions were consistent with our previous study [11]. In this study, we exposed brain slices containing the PRH cortex to THz waves at a frequency of 0.152 THz with a power output of 12.6 mW/cm^2^ for 60 min. During exposure, the slices were continuously perfused with oxygen-saturated ACSF. To prevent the ACSF from absorbing THz waves, THz waves were emitted from beneath the chamber. A THz lens (TYDEX, Russia) was employed to focus scattered light into a directional beam (Fig. S2a-b). In addition, a fibre optic temperature monitoring system (Fiso, Canada) was used to track changes in ACSF temperature during terahertz wave exposure, with the temperature ranging from 0.25 ± 0.04 °C (Fig. S3).

### Behaviour tests

All the behaviour tests were conducted according to standard protocols [7]. OFT, NOP, NLP and OiP experiments were performed in sequence after THz wave exposure. The detailed experimental methods were as follows: the NOP test consisted of a sample and a test trial. In the sample trial, the mice typically encountered two duplicates of novel objects in a habituated environment. In the test trial, the mice were presented with one previously shown object (familiar) and one new object (novel). The familiar and novel objects were placed at the same locations as in the sample trial. The novel object DI was calculated as the time spent exploring novel objects/total time (Fig. 1f). The procedure used for the NLP test was identical to that of the NOP test except that, in the test trial, one of the familiar objects was positioned at a novel location. Mice normally spend more time exploring an object at a novel location than at a familiar location. The novel location DI was calculated as the time spent exploring new location objects/total time (Fig. 1i). For the OiP test, four distinct objects located at four different locations were presented in the sample trial. After a 5 min delay, the locations of the two objects were interchanged in the test trial. The DI was calculated as the time spent in the interchanged locations/total time (Fig. 1l). To compare the terahertz effects and optogenetic modulation, the rAAV-CaMKIIα-ChrimsonR-mCherry and control viruses were injected into the PRH cortex. After 2 weeks, the NLP test was conducted. To investigate the role of PRH glutamatergic neurons in THz wave neural regulation, rAAV-CaMKIIα-hM4Di-EGFP and the control virus were injected into the PRH. After 2 weeks of viral expression, the NLP test was conducted.

### Immunohistochemistry (IHC)

The mice were anaesthetized with an overdose of pentobarbital immediately after THz stimulation and perfused intracardially with phosphate-buffered saline (PBS), followed by paraformaldehyde (PFA, 4% wt/vol in PBS). The brains were removed, fixed in 4% PFA for 4 h at room temperature, and then cryoprotected in a 30% sucrose solution until the tissues sank. Subsequently, 35 μm slices were prepared using a cryostat microtome (Leica CM1950, Germany) [17]. The samples were permeabilized in PBS with 0.3% Triton X-100 (PBST) and blocked in 3% BSA in PBST at room temperature for 1 h. The sections were then incubated with primary antibodies (anti-CaMKII alpha, 1:500, MA1-048, Invitrogen for 24 h; anti-c-Fos, 1:500, ab190289, Abcam, for 24 h; anti-VGAT, 1:200, SAB4501074, Sigma, for 24 h) at 4 °C. The sections were washed three times in PBST and were then incubated with fluorescent secondary antibodies (Alexa Fluor 488-conjugated goat anti-rabbit, 1:500; A-11008, Invitrogen; Alexa Fluor 647-conjugated goat anti-mouse, 1:500; A-21240, Invitrogen) at room temperature for 2 h, followed by three washes in PBS.

### HD-MEA

The MaxOne HD-MEA system (Maxwell, Switzerland) was used to record spontaneous and synchronous neuronal discharge in the PRH cortex. The HD-MEA system contained 26,400 electrodes and 1024 channels integrated in a 4 × 2 mm chip [18]. Baseline spontaneous activity was recorded for 5 min, followed by incubation with 10 µM bicuculline, a GABA_A_ receptor antagonist. The whole recording process was performed under perfusion of ACSF with oxygenation (95% O_2_/5% CO_2_) at 33 ± 1 °C. The spikes were detected using a bandpass filter (300–3000 Hz). Then, the synchronous discharge was recorded for 5 min, and the discharge grating image and synchronous discharge characteristics were analysed.

### Electrophysiology

Brain slices containing the PRH cortex were placed in a recording chamber that was superfused (2 ml/min) with ACSF at 32–34 °C. Neurons in the PRH cortex were observed by infrared optics using an upright fixed microscope equipped with a 40× water-immersion lens and a CCD monochrome video camera (MIGHTEX, CXE-B013-U, USA). Patch pipettes were prepared using a horizontal pipette puller (P-97, Sutter Instruments) with a resistance of 4–6 MΩ. Electrophysiological recordings were performed using a MultiClamp 700B amplifier (Molecular Devices, USA) with a Digidata 1550 A AD board (Molecular Devices, USA). The signals were filtered at 2 kHz, digitized at 10 kHz, and acquired using pClamp 10.6 software (Molecular Devices, USA). The data were analysed using Clampfit 10.6 (Molecular Devices, USA).

The action potential (AP) recording was performed using a current clamp, and APs were elicited by injecting depolarizing current steps. The pipette mixture contained 130 mM K-gluconate, 5 mM NaCl, 10 mM HEPES, 0.5 mM EGTA, 2 mM MgCl_2_, 4 mM Na-ATP, 0.4 mM Na-GTP and 10 mM phosphocreatine (pH 7.35, 290 mOsm), and AP was evoked with 500-ms-duration current pulses [19]. The total number of APs was counted and then divided by the duration of the recording period to obtain the firing rate (Hz).

For the sEPSC recording, the neurons were held at -70 mV in the presence of bicuculline (10 µM) in ACSF, with the pipette mixture containing 130 mM K-gluconate, 5 mM NaCl, 10 mM HEPES, 0.5 mM EGTA, 2 mM MgCl_2_, 4 mM Na-ATP, 0.4 mM Na-GTP and 10 mM phosphocreatine (pH 7.35, 290 mOsm). For the mEPSC recording, the NaCl in ACSF was replaced with 140 mM choline chloride.

For the sIPSC recording, the neurons were held at -60 mV in the presence of 6-cyano-7-nitroquinoxaline-2,3-dione (CNQX, 10 µM) or DL-2-amino-5-phosphonopentanoic acid (DL-AP5, 50 µM), with a pipette mixture containing 140 mM KCl, 10 mM HEPES, 4 mM MgCl_2_, 2 mM Na-ATP, 0.3 mM Na-GTP, and 0.1 mM EGTA (pH 7.35, 290 mOsm). For the mIPSC recording, the NaCl in the ACSF was replaced with 140 mM choline chloride to inhibit AP firing.

NMDAR currents were recorded at + 40 mV with bicuculline (10 µM) and CNQX (10 µM) in ACSF. Concentric stimulation electrodes were positioned equidistantly (approximately 500 μm) from the recording electrode. Stimulation was delivered at 0.03 Hz, the pulse duration was 0.1 ms, and the intensity was adjusted to elicit maximal NMDAR currents. An average of 20–30 responses was recorded. AMPAR currents were recorded at -70 mV with bicuculline (10 µM) and D-APV (50 µM) in ACSF [20].

### LTP recording

Brain slices containing the PRH cortex were submerged, perfused with ACSF and transferred to the recording chamber. The recording electrode (1–2 MΩ) filled with ACSF was placed into layer II or layer III of the PRH cortex. Concentric stimulation electrodes were positioned equidistantly (approximately 500 μm) from the recording electrode, one in the anterior direction and one in the posterior direction [21]. The electrode depth, stimulation intensity, and positioning of both electrodes were adjusted to optimize the maximum fEPSP amplitude. The stimulation intensity was subsequently reduced to 50%, and a 30 min baseline recording was obtained. Electrical stimulation was delivered using a stimulator (AM SYSTEM, USA) with a pulse duration of 0.1 ms, and LTP was induced by applying three stimulations (1 s high-frequency stimulation trains at 100 Hz with 20 s intervals among each stimulation). After high-frequency stimulation, fEPSPs were recorded for 50 min. During both the baseline and post-HFS periods, stimulation was delivered every 30 s. For clarity, in the time-course plot of fEPSP slopes, one representative data point was plotted every 5 min.

### In vitro calcium imaging

The mice were anaesthetized with isoflurane. rAAV-CaMKIIa-jGCaMP6f (Brain Case, China) was injected into the PRH cortex (AP: -1.7 mm, ML: ±4 mm, DV: 4 mm). After 2 weeks, the calcium signals in the brain slices were recorded using a CCD camera (MIGHTEX, USA). The fluorescent calcium indicator was excited at a wavelength of 470 nm. A brain slice was placed in a perfusion chamber and continuously perfused with oxygenated ACSF at a constant flow rate of 2 ml/min. Neuronal calcium signals induced by electrical stimulation (100 Hz, 0.1 mA, 0.45 ms, 2 s) have been described previously [22], and time series images of calcium signals were captured using a high-speed CCD camera. The fluorescence images were analysed with IAA software (MIGHTEX, USA), and the changes in the calcium signal are expressed as ΔF/F, where ΔF represents the change in fluorescence intensity and F represents the fluorescence intensity at each time point.

### Western blot (WB) analysis

Proteins were extracted from mouse brain sections containing the PRH cortex. Lysates were analysed on the JESS Simple Western™ instrument (Protein Simple, USA) [23], with the addition of 5 µL per well of primary antibodies (anti-VAMP2, 1:500, ab181869, Abcam; anti-synaptophysin, 1:1000, ab32127, Abcam; anti-Syntaxin1, 1:200, ab224223, Abcam; anti-GAPDH, 1:500, ab181602, Abcam; anti-GluN1, 1:50, ab109182, Abcam; anti-GluN2A, 1:50, ab124913, Abcam; anti-GluN2B, 1:50, ab254356, Abcam; anti-GluA1, 1:50, ab31232, Abcam; anti-GluA2, 1:50, 13607s, CST; anti-glutamine synthetase, 1:100, ab16802, Abcam; anti-glutaminase, 1:100, 822203864, Genetex; anti-EAAT1, 1:50, 5684 S, CST; anti-EAAT2, 1:50, ab41621, Abcam; anti-CaMKII, 1:50, ab134041, Abcam; anti-PSD95, 1:100, ab238135, Abcam). Overall, the manufacturer’s protocol was followed with modifications only to the volumes applied. The secondary antibody (anti-rabbit HRP or anti-mouse HRP) and enhanced chemiluminescence (ECL) reagents were used according to the instructions supplied with the kit (anti-rabbit detection module chemiluminescence; ProteinSimple^®^, Bio-Techne). The antibody diluents, washing buffers, plates and capillary cartridges used were derived from the 12–230 kDa separation module (ProteinSimple^®^, Bio-Techne). After the experiment, the results were analysed using Compass for Simple Western software. Signal detection was performed using chemiluminescence, and the protein bands were visualized accordingly. The relative protein expression levels were calculated and compared across different experimental groups. The exposure times were adjusted to ensure optimal signal detection without oversaturation. All the experiments were repeated at least three times to ensure reproducibility, and statistical analysis was performed to confirm significant differences between conditions.

### Optogenetic inhibition experiment combined with AP recording

rAAV-CaMKII-ArchT-EGFP and rAAV-CaMKII-EGFP (Brain Case, China) were injected into the PRH cortex (AP: -1.7 mm, ML: ±4 mm, and DV: 4 mm). After 2 weeks, the brain sections were made to record AP. The groups were divided as follows: the control group, which was injected with the control virus without terahertz exposure (C group); the terahertz group, which was injected with the control virus and exposed to terahertz waves (T group); the control group, which was injected with the optogenetic inhibition virus without terahertz exposure (C+Light group); and the terahertz group, which was injected with the inhibition virus and exposed to terahertz waves (T+Light group). The C+Light group and T+Light group were subjected to 562 nm photostimulation using an in vitro optogenetic system (MIGHTEX, USA). The laser power was set to 10 mW, and each stimulation lasted for 120 s. Simultaneously, the AP recordings of neurons in the PRH region were performed using a MultiClamp 700B amplifier. Patch clamp sealing and recording were performed following standard protocols that have been described previously. The number and frequency of APs at 400 pA were quantitatively analysed.

### Statistical analysis

SPSS 10.0 software was used for analysis via the independent samples t test and one-way ANOVA with 95% confidence intervals. The data are reported as the means ± SEMs in all figures. The accepted level of significance was a P value < 0.05. Detailed statistical information is provided in the supplementary table.

## Results

### Terahertz waves improved NLP-mediated visual recognition memory

One week after skull transparency, the mice were subjected to three days of habituation to minimize anxiety caused by the presence of the experimenters. Sixty minutes after THz wave exposure, the open field test (OFT) and NOP experiments were conducted (Fig. 1b). There was no significant change in the exploration time or distance travelled by the mice in the centre area, indicating that THz wave exposure had no significant effect on the locomotor activity or exploratory behavior of the mice (Fig. 1c-e). For the NOP test, the exploratory time and discrimination index (DI) did not significantly differ between the two groups (Fig. 1f-h). The DI of the mice in the terahertz exposure group increased significantly during the NLP test, indicating that terahertz waves improved NLP-mediated visual recognition memory (Fig. 1i-k). In addition, there were no significant differences during the OiP test (Fig. 1l-n).

**Fig. 2:**
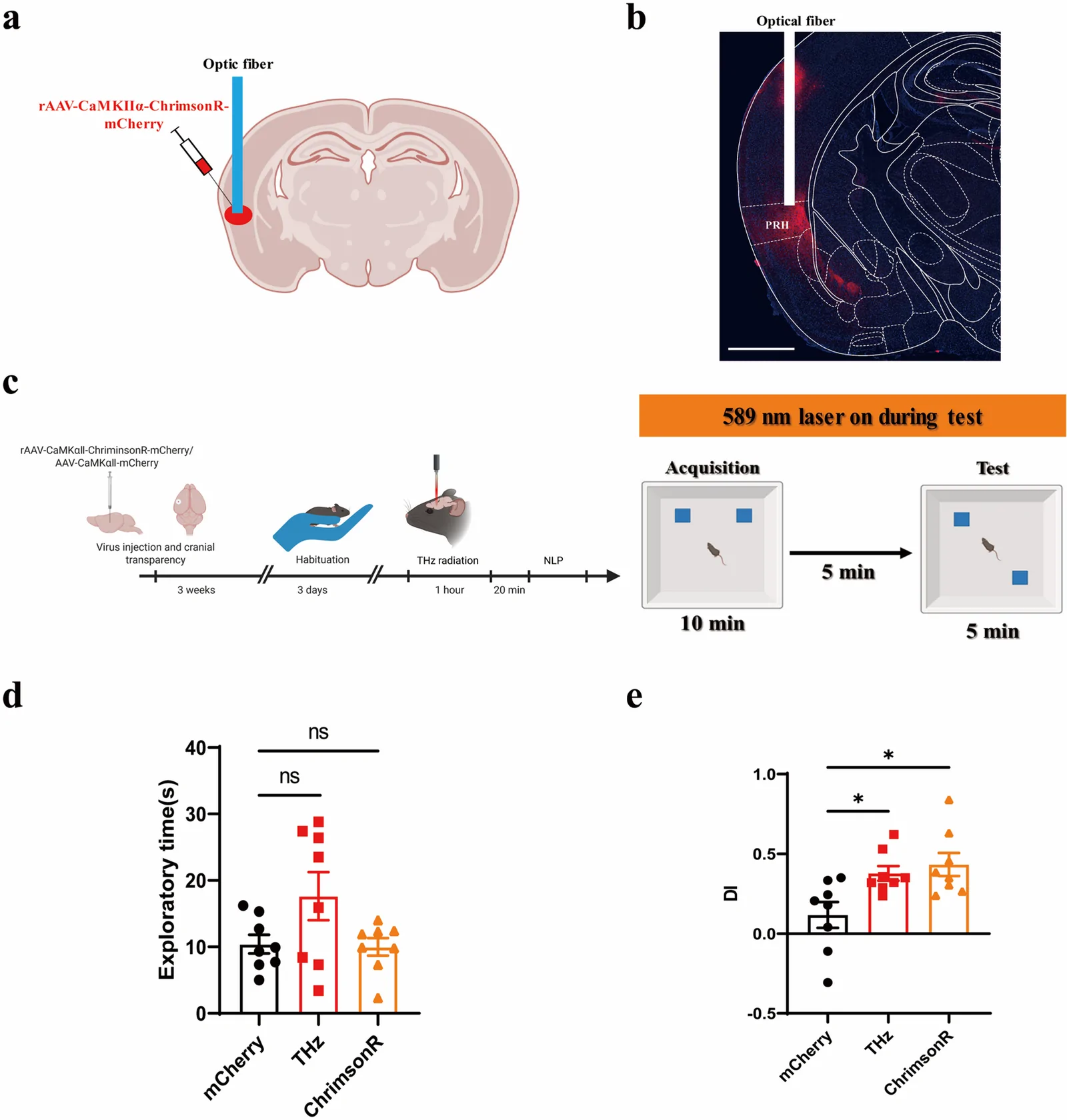
Terahertz waves and optogenetic enhanced NLP mediated visual recognition memory. **a** Schematic of PRH injected an optogenetic virus. **b** An image showing CaMKIIα-ChrimsonR-mCherry (red) and immunohistochemical staining for DAPI (blue), Scale bar = 1000 μm. **c** Flowchart of cranial transparency surgery and NLP with optogenetic and the light exposure strategy during NLP in mice. **d**-**e** Effects of optogenetic activation of glutamatergic neurons in PRH on object location memory (*n* = 8 mice). All data are presented as mean ± SEM. The significance was assessed using unpaired *t*-test. *p < 0.05, ns (not significant)

Given the necessity of the PRH cortex in regulating recognition memory, we found that terahertz waves could improve location-related memory. We also compared the behavioural effects of THz waves and optogenetics. Compared with that of the mCherry group, the DI of the mice in the NLP test increased significantly between the THz and ChrimsonR groups (Fig. 2a-e).

**Fig. 3:**
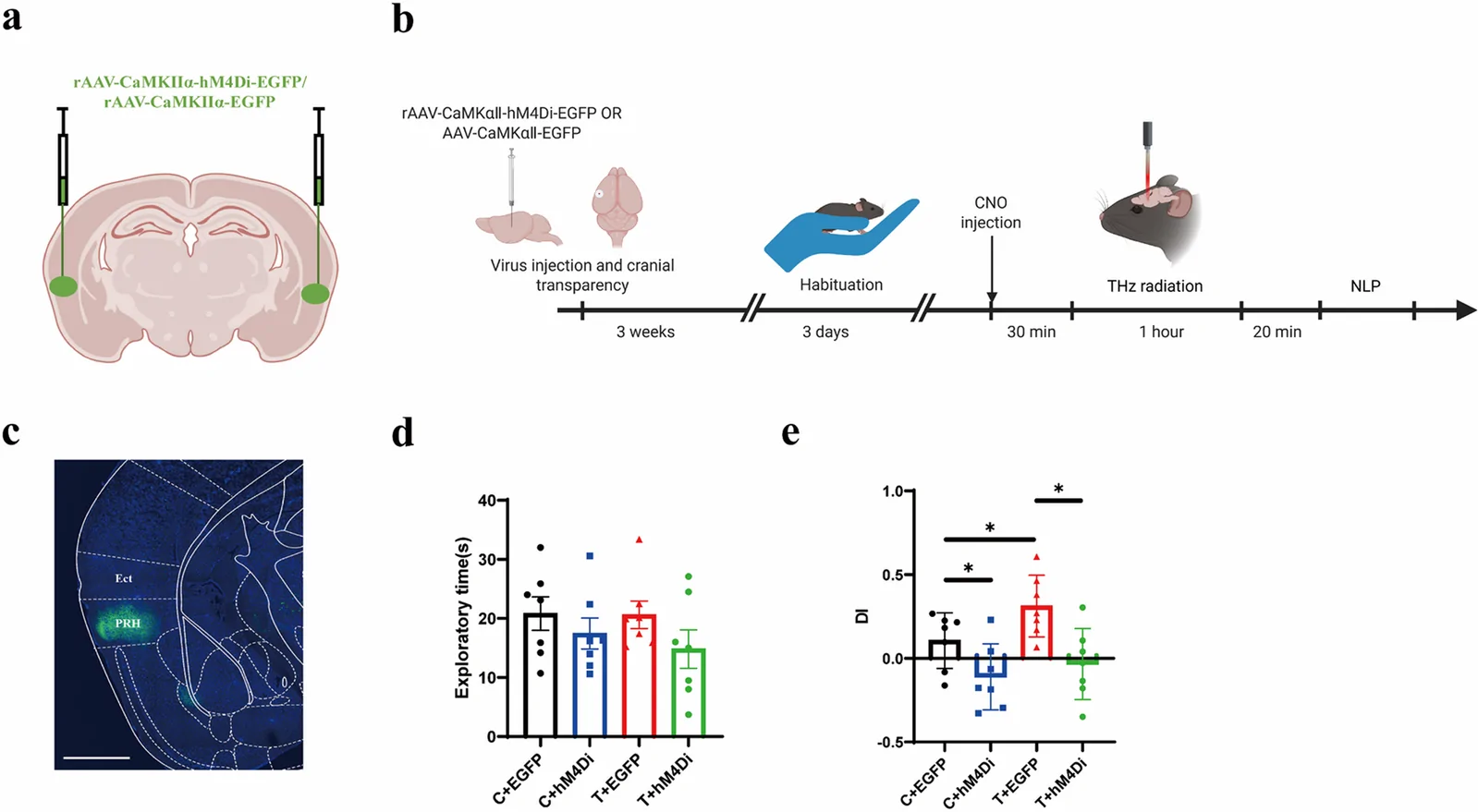
Terahertz waves and Chemical genetics intervened NLP mediated visual recognition memory. **a** Schematic of PRH and PRH injected Chemical genetics virus. **b** Schematic diagram of the behavioral process combining THz wave radiation with chemogenetics. **c** An image showing CaMKII-DIO-hM4Di-EGFP (green) and immunohistochemical staining for DAPI (blue), Scale bar = 1000 μm. **d**-**e** Effects of Chemical genetics inhibition of glutamatergic neurons in PRH on object location memory (*n* = 9 mice). All data are presented as mean ± SEM. The significance was assessed using unpaired *t*-test. *p < 0.05, ns (not significant)

We found that THz waves could improve location recognition memory, similar to the optogenetic method of activating glutamatergic neurons in the PRH. In addition, after chemical genetic inhibition of these neurons, the regulatory effect of THz wave exposure on mouse behaviour was suppressed, indicating that THz waves augmented the visual recognition memory of mice by enhancing the function of PRH glutamatergic neurons (Fig. 3a-e).

### Terahertz waves increase neuronal activity, excitability and synaptic plasticity in PRH

**Fig. 4:**
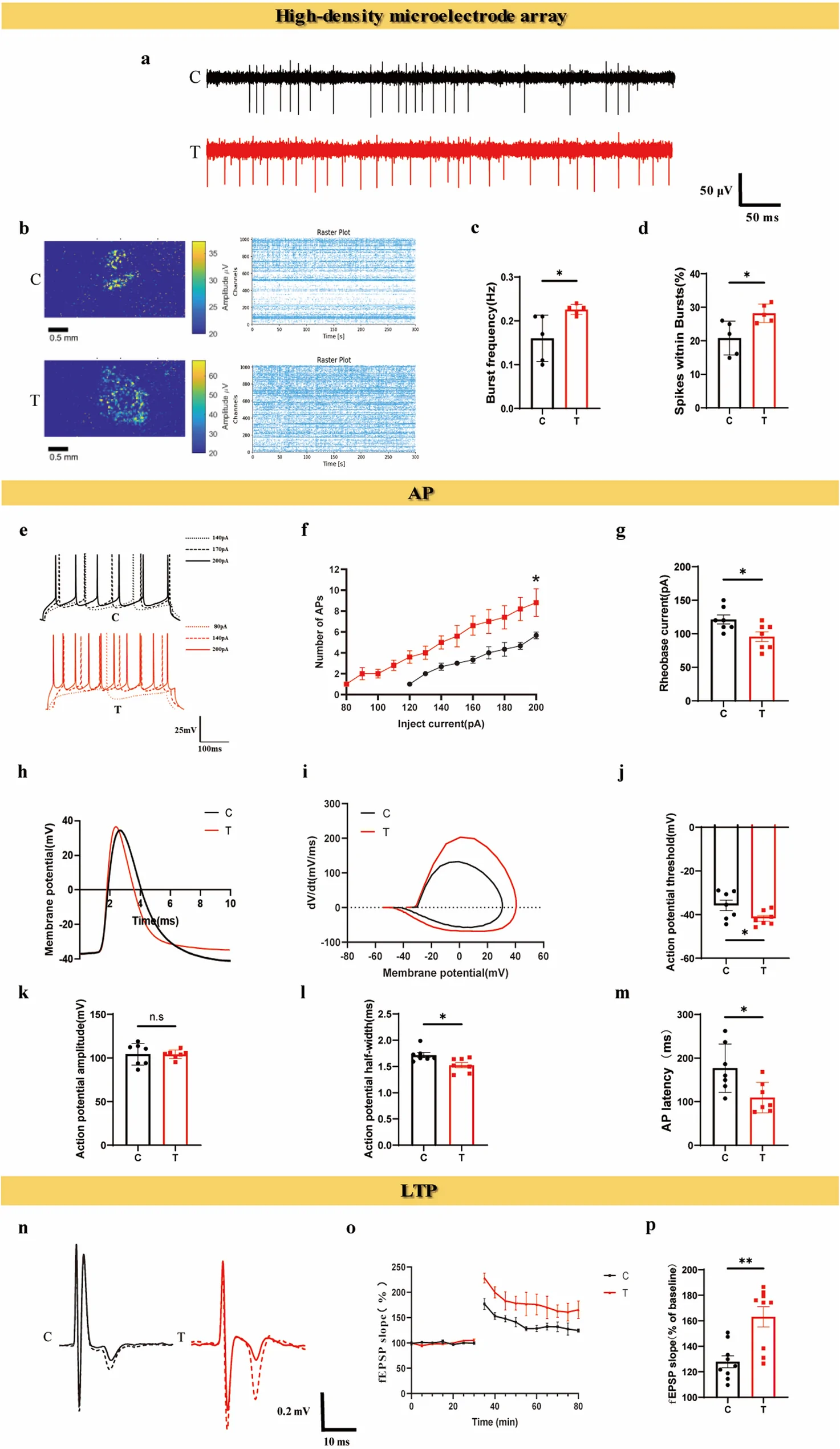
Enhanced neuronal firing, synaptic functional plasticity and excitation in the PRH after THz wave exposure. **a** Single traces of bursting activity in the C and T groups. **b** Heatmaps and raster maps of the synchronous firing amplitude of neurons in the PRH; scale bar = 0.5 mm. **c**-**d** Changes in neural spike firing in the PRH after THz wave radiation (*n* = 5 brain slices). **e** Three representative traces of APs evoked by the 0–200-pA injected current. **f** Curves of the number of APs in neurons evoked by injections of inwards currents (*n* = 5 cells). **g** Analysis of the AP rheobase current between the C and T groups (*n* = 7 cells). **h** Representative trace of a single AP evoked by the injection of a rheobase current. **i** Plots of the neuronal membrane potential versus dv/dt during the AP. **j**-**m** Changes in the neural AP threshold, amplitude, half-width and latency between the C and T groups (*n* = 7 cells). **n** Representative traces of fEPSPs after basic (dotted line) and high-frequency (solid line) electrical stimulation in the PRH. **o** Changes in the fEPSP slope in the PRH during electrical stimulation (*n* = 3 brain slices; 0–30 min: basic stimulation; 30–80 min: high-frequency stimulation). **p** Comparison of the mean fEPSP slopes at 50–80 min between the C and T groups (*n* = 9 cells). All the data are presented as the means ± SEMs. The significance was assessed using an unpaired *t* test. *p < 0.05, **p < 0.01, ns (not significant)

To evaluate the effects of terahertz waves on neuronal activity, a high-density microelectrode array (HD-MEA) was used to record extracellular firing activity in the PRH (Fig. 4a). Heatmaps and raster plots were generated, indicating increased spontaneous neuronal firing after terahertz exposure (Fig. 4b). Moreover, the burst frequency was compared between the C and T groups. A significantly increased burst frequency was observed in the T group (Fig. 4c). We also found that the proportion of spikes within bursts increased after THz exposure (Fig. 4d). These results indicated that THz exposure enhanced neuronal activity in the PRH. The excitability of neurons was assessed by AP. The number of APs evoked by step currents from 0 to 200 pA significantly increased, and the rheobase current (the minimum current required to excite the AP) decreased after THz radiation (Fig. 4e-g). The single AP showed dramatic waveform changes (Fig. 4h, i). The AP threshold significantly decreased, and the half-width of the AP and the AP latency (the time from the onset of current injection to the generation of the first AP) dramatically decreased in the T group (Fig. 4j-m). Together, the results revealed increased excitability of neurons in the PRH after THz wave exposure. Furthermore, functional synaptic plasticity was assessed by LTP recording. Compared with that in the C group, the field excitatory postsynaptic potential (fEPSP) slope at 30–80 min was significantly increased in the T group (Fig. 4n-p), indicating that THz wave exposure enhanced the functional synaptic plasticity of neurons in the PRH.

### THz waves increase the excitability of glutamatergic neurons in the PRH

**Fig. 5:**
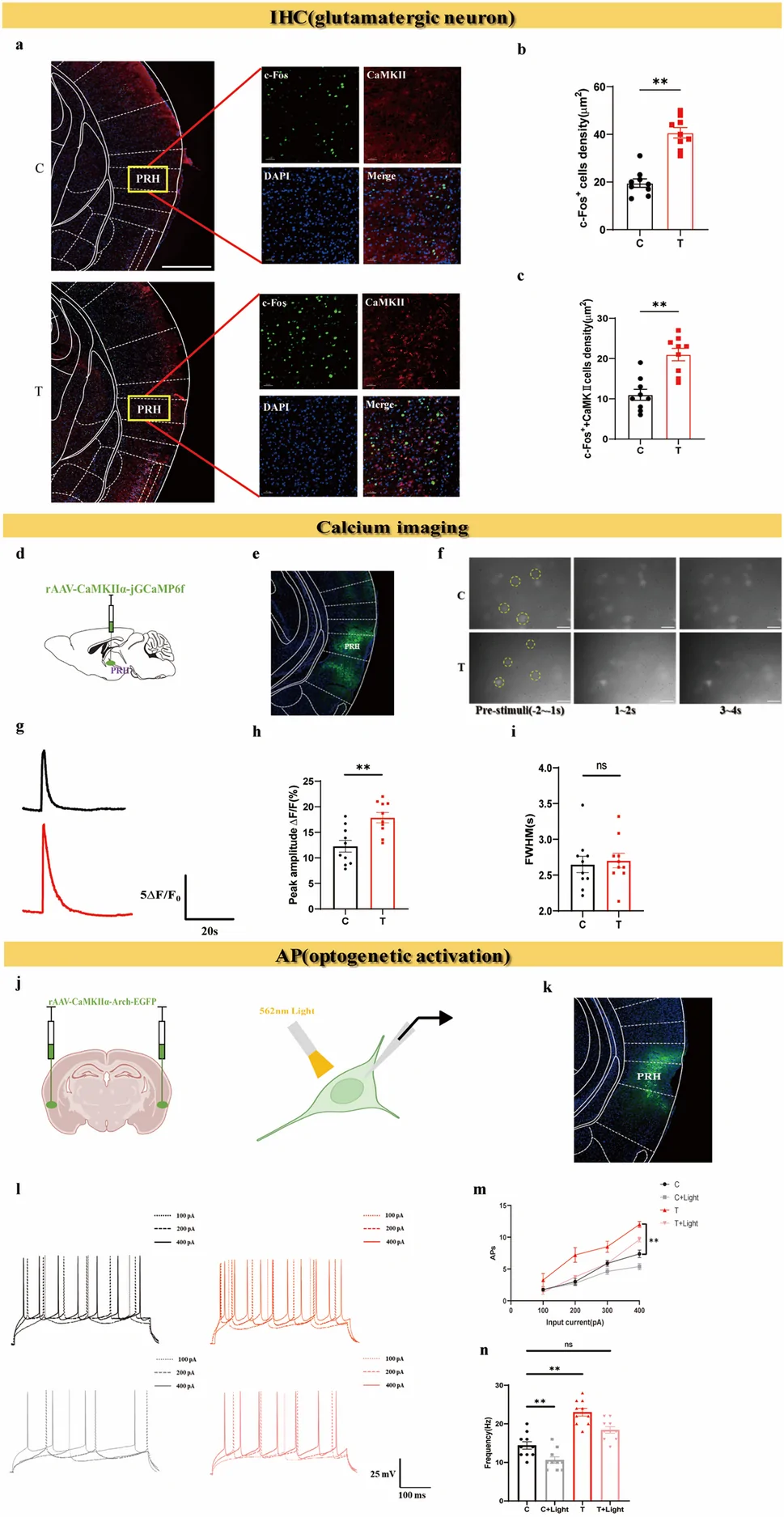
Enhanced excitability of glutamatergic neurons in the PRH after THz wave exposure. **a** Representative images showing the distribution of c-Fos^+^ (green) and CaMKⅡα^+^ (red) colabelled neurons in the PRH; scale bars = 1000 μm (left) and 50 μm (right). **b**-**c** The effects of THz waves on glutamatergic neuronal activity (*n* = 9 brain slices). **d** Schematic of injecting calcium imaging virus into the PRH. **e** An image showing CaMKIIα-jGCaMP6f-EGFP (green) and immunohistochemical staining for DAPI (blue). Scale bar = 1000 μm. **f** Real-time plots of calcium signal changes in glutamatergic neurons in the PRH during electrical stimulation; scale bar = 10 μm. **g** Representative traces of the calcium signal peak in the C and T groups. **h**-**i** Effects of THz waves on the calcium signals of glutamatergic neurons in the PRH (*n* = 10 cells). **j** Schematic of PRH-injected virus combined with whole-cell recording. **k** An image showing CaMKIIα-ArchT-EGFP (green) and immunohistochemical staining for DAPI (blue). Scale bar = 1000 μm. **l** Representative traces of APs with 0–400-pA injected currents during 562-nm light stimulation. **m** Changes in the AP number of neurons evoked by injections of inwards currents (*n* = 8 cells). **n** Effects of THz waves on the firing rate of glutamatergic neurons in the PRH (*n* = 10 cells). All the data are presented as the means ± SEMs. The significance was assessed using an unpaired *t* test. *p < 0.05, **p < 0.01, ns (not significant)

Researchers have reported that the LTP of the PRH is a specific input that relies on the NMDA receptor and displays associative properties [9, 21]. The increased fEPSP slope during LTP indicates that terahertz irradiation might affect glutamatergic neurons or ionic glutamate receptors. Therefore, we assessed the effects of THz waves on glutamatergic neurons. First, immunofluorescence staining for CaMKIIα and c-Fos was conducted to label activated glutamatergic neurons (Fig. 5a). The number of c-Fos-positive neurons increased significantly in the T group. Moreover, the number of neurons colabelled with both c-Fos and CaMKIIα also increased, indicating that THz waves improved the activation of glutamatergic neurons (Fig. 5b, c). Next, we employed calcium imaging to record neuronal activity after THz wave irradiation. Neurons in the PRH were labelled with an adeno-associated virus named rAAV-CaMKIIα- jGCaMP6f (Fig. 5d-f). Electrodes were used to stimulate Layer 2/3 in the PRH to evoke calcium transients [22], which were recorded at an excitation wavelength of 470 nm. An increased peak amplitude was found in the T group (Fig. 5g-i), indicating that THz waves could enhance the calcium activity.

To confirm the excitability of glutamatergic neurons after THz wave exposure, we used an optogenetic protocol to inhibit the activity of glutamatergic neurons (Fig. 5j-k) and recorded the changes in APs (Fig. 5l). A comparison of the C and C+Light groups revealed that the virus (rAAV-CaMKIIα-ArchT-EGFP) significantly inhibited the firing of APs. After the inhibition of glutamatergic neuron activity, the improvement effect of THz waves on APs was suppressed (Fig. 5m-n). These results indicated that THz waves improved neuronal excitability and that glutamatergic neurons contributed to the neural enhancement effect of THz waves in the PRH.

**Fig. 6:**
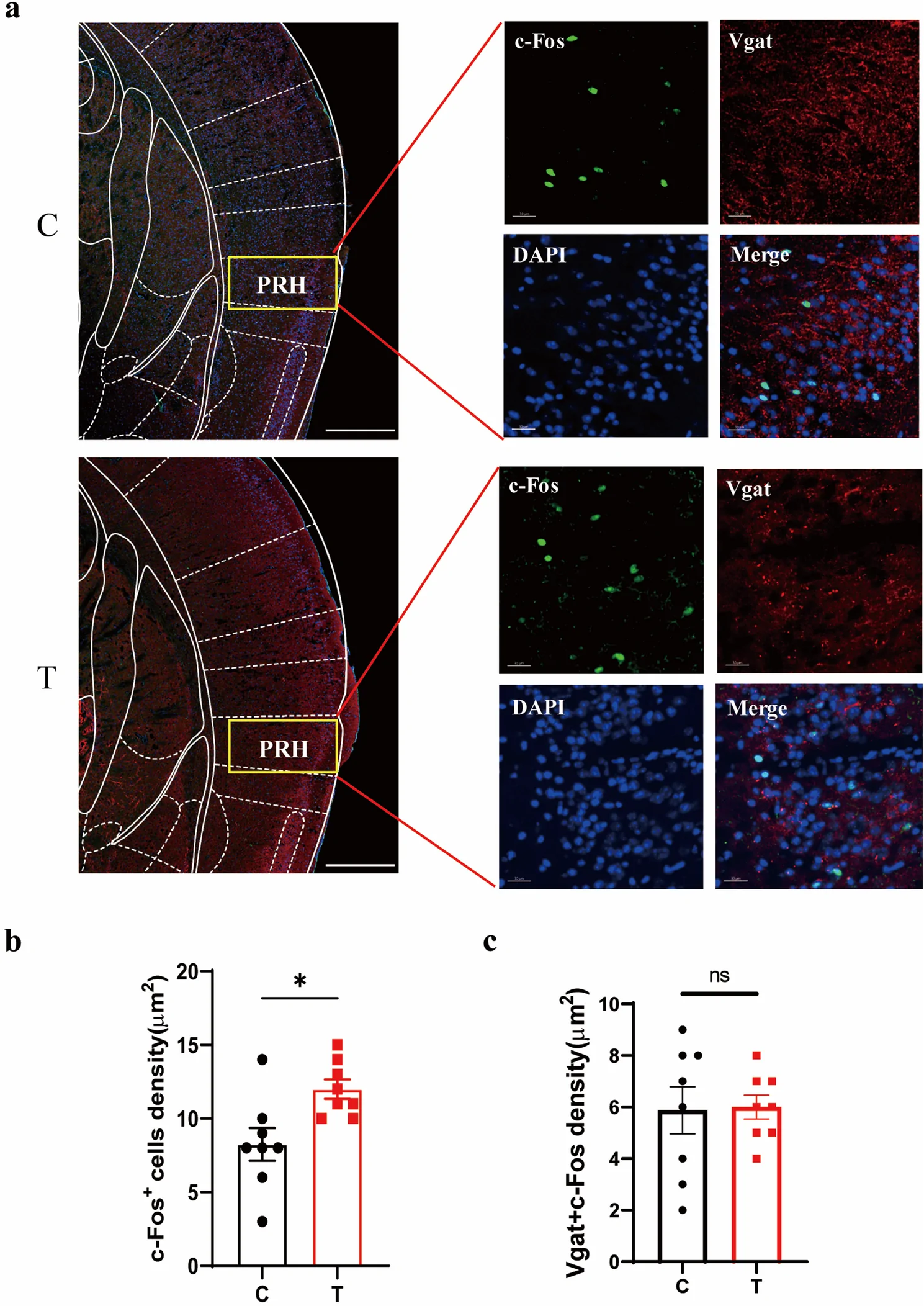
The excitability of GABAergic neurons in the PRH did not change significantly after THz wave exposure. **a** Representative images showing distribution of c-Fos^+^ (green) and VGAT^+^ (red) co-labelled neuron in PRH, scale bar = 1000 μm (left), 50 μm (right). **b**-**c** Fos^+^ cells density and c-Fos^+^/VGAT^+^ cells density (*n* = 8 brain slices). All data are presented as mean ± SEM. The significance was assessed using unpaired *t*-test. *p < 0.05, ns (not significant)

In addition, we performed immunofluorescence staining with VGAT and c-Fos to label activated GABAergic neurons. The number of c-Fos-positive neurons was significantly increased in the T group. However, the number of neurons labeled with both c-Fos and VGAT did not change significantly, indicating that terahertz waves have no effect on the activity of GABAergic neurons. (Fig. 6a-c).

### THz waves accelerate glutamate transmission in PRH

**Fig. 7:**
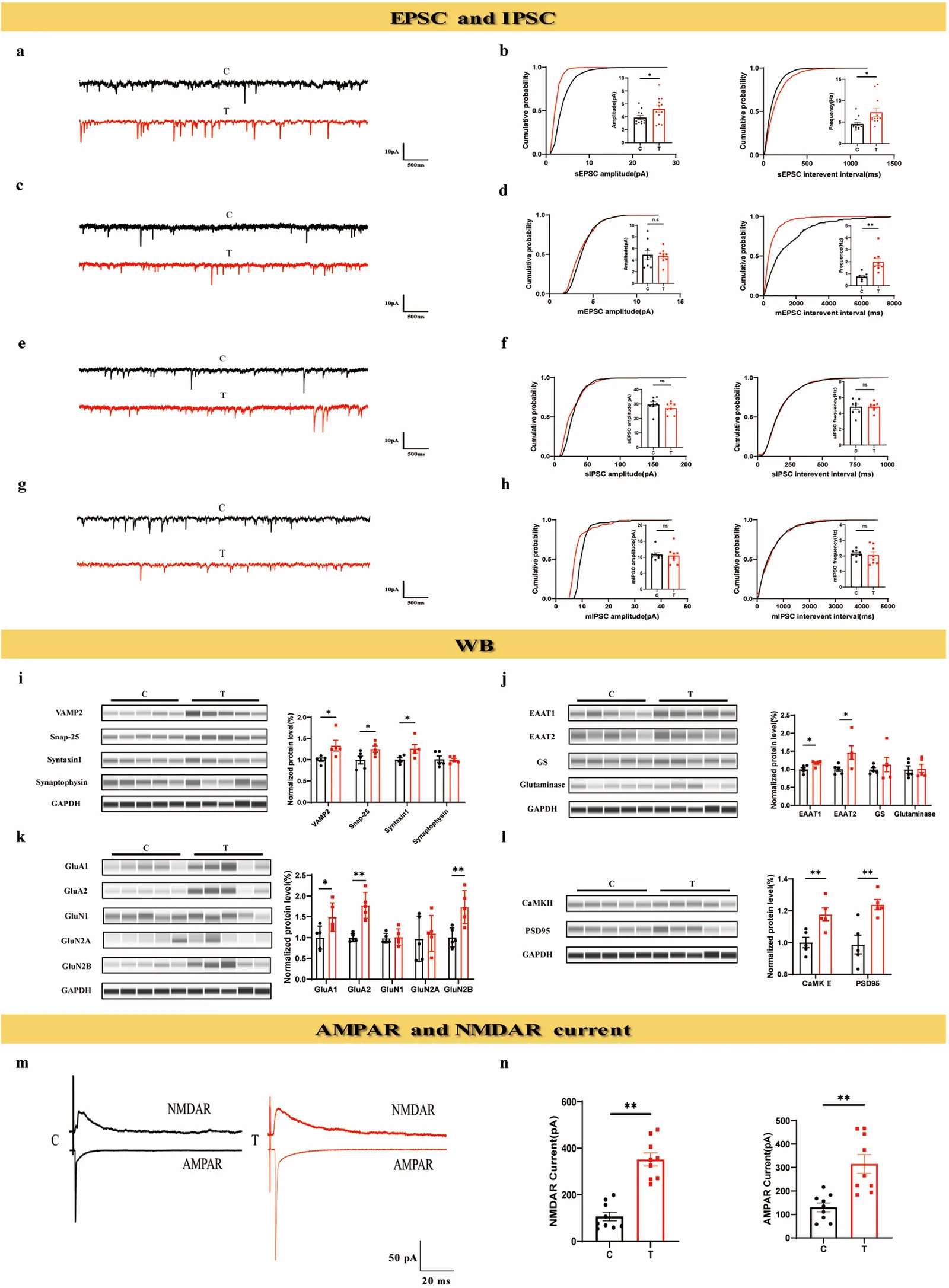
Accelerated glutamate transmission in PRH neurons after THz wave exposure. **a** Representative traces of sEPSCs (C group: black; T group: red). **b** Cumulative probability curves for the amplitude (left) and interevent interval (right) distributions and data analysis of sEPSCs (*n* = 12 cells). **c** Representative traces of mEPSCs. **d** Cumulative probability curves for the amplitude (left) and interevent interval (right) distributions and data analysis of mEPSCs (*n* = 9 cells). **e** Representative traces of sIPSCs. **f** Cumulative probability curves for the amplitude (left) and interevent interval (right) distributions and data analysis of sIPSCs (*n* = 9 cells). **g** Representative traces of mIPSCs. **h** Cumulative probability curves for the amplitude (left) and interevent interval (right) distributions and data analysis of mIPSCs (*n* = 9 cells). **i** Analysis of vesicle release protein expression in the PRH (*n* = 5 brain slices). **j** Analysis of glutamate-metabolizing protein expression in the PRH (*n* = 5 brain slices). **k** Analysis of glutamate receptor subunit expression in the PRH (*n* = 5 brain slices). **l** Analysis of postsynaptic protein expression in the PRH (*n* = 5 brain slices). **m** Representative traces of AMPAR and NMDAR currents in the C and T groups. **n** Analysis of AMPAR and NMDAR currents in the PRH (*n* = 9 cells). All the data are presented as the means ± SEMs. Significance was assessed using an unpaired *t* test. *p < 0.05, **p < 0.01, ns (not significant)

To determine the effects of THz waves on synaptic transmission, we recorded changes in excitatory postsynaptic currents (EPSCs) and inhibitory postsynaptic currents (IPSCs). The amplitude and frequency of spontaneous excitatory postsynaptic currents (sEPSCs) increased significantly in the terahertz group (Fig. 7a, b). The frequency of miniature excitatory postsynaptic currents (mEPSCs) increased significantly after THz exposure (Fig. 7c, d). However, there were no significant changes in spontaneous inhibitory postsynaptic currents (sIPSCs) or miniature inhibitory postsynaptic currents (mIPSCs) after THz exposure (Fig. 7e-h). These results indicated that THz waves could mainly accelerate excitatory synaptic transmission.

Researchers have reported that glutamatergic neurotransmission plays important roles in excitatory synaptic transmission [24]. Many types of transporters contribute to glutamatergic excitatory synaptic transmission. First, glutamate is loaded into synaptic vesicles by vesicular glutamate transporters to be released from presynaptic terminals. After synaptic vesicle release, glutamate is taken up by neurons or astrocytes to terminate the signal and prepare for the next signal [25]. Therefore, we detected changes in molecules related to glutamate metabolism.

The soluble N-ethylmaleimide-sensitive factor attachment protein receptor (SNARE) complex forms tight complexes that are crucial for membrane fusion and neurotransmitter release [26]. Vesicle-associated membrane protein 2 (VAMP2), syntaxin-1, synaptosomal-associated protein 25 (Snap-25), and synaptophysin (Syp) are important components of the SNARE complex at the presynapse and play crucial roles in modulating neurotransmitter release, vesicle trafficking, and synaptic plasticity [27–29]. The levels of VAMP2, syntaxin-1 and snap-25 significantly increased after THz wave exposure, indicating an increase in neurotransmitter release ability (Fig. 7i). The glutamate‒glutamine cycle is important for maintaining the normal level of glutamate in the synaptic cleft [30]. We found that the levels of excitatory amino acid transporters 1–2 (EAAT1 and EAAT2) increased significantly, indicating the enhanced clearance ability of glutamate (Fig. 7j). The α-amino-3-hydroxy-5-methyl-4-isoxazole-propionic acid receptor (AMPAR) and N-methyl-D-aspartate receptor (NMDAR) were important ionic glutamate receptors. The subunits of AMPARs and NMDARs also increased (Fig. 7k). Moreover, increased expression of the postsynaptic molecules CaMKII and PSD95 was detected in the terahertz group (Fig. 7l). In addition, AMPAR and NMDAR currents also increased after THz wave exposure (Fig. 7m-n). The above results indicated that THz waves could induce accelerated glutamate transmission, which was consistent with the increase in EPSCs.

## Discussion and conclusion

An increasing number of studies have focused on the interaction of THz waves with the nervous system [1, 12, 31]. Compared with common optical modulation methods, THz waves, which have the advantages of low photon energy and a reduced risk of phototoxicity or tissue damage, could serve as a promising new approach for neuromodulation, referring to the regulation of neuronal activity through external stimulation. Scientists have performed many in vivo animal experiments and found that THz waves can change behaviour. Increased levels of anxiety and depression were found in albino rats subjected to acute immobilization stress after intensive terahertz irradiation [32, 33]. The 3.6 THz waves can increase anxiety in male mice [34]. However, THz waves (0.14 THz for 20 min per day, 2 weeks) might increase antianxiety, antidepression, and social interaction effects in mice [35]. In higher frequency bands, THz waves have been found to improve learning and memory abilities and associative learning, but as the temperature increases to over 1 °C [6, 36]. In our study, we focused on the lower frequency of THz bands and avoided significant temperature increases. The PRH is also an important brain area for recognition memory. We found that 0.152 THz waves could significantly improve visual recognition memory. Interestingly, while 0.152 THz waves significantly enhanced performance in the NLP test, no such effect was observed in the OiP test. This discrepancy likely stems from the differing cognitive demands of the two tasks. The OiP test involves complex object-place associations among four items, requiring a higher level of integrative network activity across multiple brain regions. In contrast, NLP is a simpler spatial recognition task. Our findings suggest that local THz stimulation in the PRH provides sufficient neuromodulation for enhancing basic spatial location memory, but may not meet the threshold required to improve more complex, multi-region associative memory tasks.

Optogenetics, which genetically targets light-sensitive opsins to specific neuronal subtypes, provides a high degree of cellular and circuit specificity. This precise control allows the selective activation or inhibition of particular neuronal populations, thereby producing well-defined behavioural and physiological outcomes. In contrast, THz waves do not require genetic modification but rely on their physical interactions with neuronal tissue. While this characteristic offers advantages in clinical and translational applications, it may limit selectivity for specific neuronal subtypes. Nonetheless, our findings indicated that 0.152 THz waves could modulate behaviour without the need for genetic manipulation, demonstrating a predominant effect on glutamatergic neurons, as evidenced by c-Fos expression, calcium imaging, and patch clamp recordings. Future investigations should examine the specificity of THz wave stimulation and determine whether varying frequencies or intensities can achieve neuromodulatory selectivity comparable to that of optogenetic approaches. Furthermore, chemogenetic inhibition experiments revealed that inhibiting PRH glutamatergic neurons abolished the behavioural enhancement effects of THz waves. These findings suggested that THz waves could improve visual recognition memory by enhancing the function of PRH glutamatergic neurons, providing new insights for future applications of THz neuromodulatory effects.

Normal neuronal activity, excitability and synaptic plasticity, activity-dependent changes in neuronal connection strength, have long been considered important components of memory [37]. Glutamate receptor plasticity in the PRH cortex is crucial for the acquisition of recognition memory [24, 38]. In this study, we first detected neuronal activity using an MEA assay and detected an increased burst frequency in the terahertz group. Moreover, the number of APs in the T group significantly increased with decreasing rheobase current. The results indicated that increased neuronal activity and excitability were observed after THz wave exposure. LTP recording also revealed increased synaptic plasticity. Recordings of electrical signals at different levels revealed that THz waves might increase the excitability of individual neurons, thereby affecting overall activity and plasticity. The sustained enhancement observed in both fEPSP slopes and behavioral discrimination indices does not imply irreversibility in a harmful sense. Instead, it reflects the successful induction of LTP, a fundamental mechanism of synaptic plasticity and memory formation. c-Fos and Fos-related antigens were used as traditional markers of neuronal activity [39]. We found that the number of c-Fos-positive cells increased after THz wave exposure, especially among CaMKII-positive neurons, which indicated that excitatory neurons or glutamatergic neurons were activated. These results suggested that THz waves might have a positive effect on memory-related neural activity by increasing the activity of glutamatergic neurons and enhancing synaptic plasticity. However, the underlying mechanism requires further study. Therefore, excitatory neurons or glutamatergic neurons merit special attention in future studies.

GCaMP6f, a common calcium indicator, was used in this study to achieve calcium imaging in glutamatergic neurons with a CaMKII promoter [40]. Researchers have reported that the application of infrared neural stimulation induces intracellular calcium signals in Layer 2/3 neurons in the mouse cortex in vivo [41]. In our study, 0.152 THz waves generated effects similar to those of infrared stimulation, broadening the application of THz waves in the neuroscience field. Moreover, the optogenetic stimulation protocol used in our study revealed that the inhibition of glutamatergic neurons could decrease THz wave-induced AP enhancement. This result confirmed that THz waves could affect mainly glutamatergic neurons, which was consistent with previous c-Fos results. In addition, 42.5 THz (7.05 μm, 1418 cm^− 1^) waves were reported to increase Ca^2+^ conductance and increase the calcium influx of Ca_V_1.2 in HEK293 cells. Furthermore, this irradiation upregulates critical Ca_V_1 signals, including CREB phosphorylation and c-Fos expression [42]. Compared with our results, we discovered similar neural activation effects at lower THz bands under nonthermal conditions.

Previous studies have focused on the neural excitability or activation effects of THz waves [4, 31, 43, 44]. In recent years, researchers have focused on the effects of THz waves on synaptic transmission and neuronal firing activities and reported that 30–45 THz waves can increase postsynaptic currents in vitro [5]. However, at lower frequencies, studies on synaptic transmission are not sufficient. Considering the important role of glutamatergic neurons, we conducted a patch clamp to record EPSCs and IPSCs to clarify the effects of THz waves on synaptic transmission. The frequencies of mEPSCs and sEPSCs significantly increased after THz wave exposure, whereas those of the IPSCs did not change. The results indicated that 0.152 THz waves could increase excitatory synaptic transmission. Next, we analysed the expression levels of important molecules involved in the metabolic process of excitatory amino acid transmission. The number of molecules involved in neurotransmitter release and vesicle trafficking increased significantly. Moreover, the subunits and currents of ion glutamate receptors also increased, which could ultimately enhance excitatory synaptic transmission and was consistent with the increase in EPSCs. This study found that terahertz waves have a dual regulatory effect on synaptic transmission. The increased frequency of mEPSCs, combined with the upregulation of SNARE proteins, indicates an enhanced presynaptic release mechanism. Simultaneously, the increased amplitude of sEPSCs, along with the enhanced expression of glutamate receptor subunits and PSD95, demonstrates enhanced postsynaptic receptor sensitivity. Taken together, these data suggest that 0.152 THz waves promote a comprehensive strengthening of synaptic connections, which constitutes the physiological basis for the observed improvement in visual recognition memory.

While acute electrophysiological changes occur within minutes, the downstream molecular consolidation, including c-Fos induction, glutamate receptor upregulation, and SNARE/PSD95/CaMKII accumulation, depends on de novo protein synthesis over tens of minutes to hours [45]. Sustained glutamatergic activation is also required to trigger NMDA receptor-dependent calcium signalling and CaMKII autophosphorylation, a molecular switch for LTP induction in the PRH [46]. Brief stimulation likely produces only subthreshold activity insufficient to engage these cumulative, threshold-dependent cascades, consistent with the inefficacy of shorter exposures [11]. From a mechanistic perspective, the requirement for prolonged exposure distinguishes the low-frequency, non-thermal THz neuromodulation paradigm from thermal effects: thermal mechanisms typically produce rapid, non-specific cellular responses that do not exhibit the cumulative, threshold-dependent kinetics observed here. Therefore, 0.152THz mainly exerts neuromodulatory effects through non-thermal effects.

Although this study provides multi-level evidence for the memory-enhancing effects of 0.152 THz waves, several limitations remain to be addressed. All electrophysiological recordings, including AMPAR and NMDAR currents, were performed after THz exposure rather than during the irradiation. Simultaneous “real-time” recording would provide more direct evidence of how THz photons instantaneously modulate ion channel kinetics. However, such experiments are currently limited by technical challenges, such as spatial constraints between THz focusing elements and patch clamp manipulators, as well as potential electromagnetic interference. Future studies should focus on developing specialized recording chambers to facilitate real-time monitoring of synaptic currents during THz stimulation. Additionally, exploring a broader range of frequencies and intensities will be essential to define the optimal parameters for nonthermal neuromodulation and to further clarify the underlying biophysical mechanisms. Furthermore, all behavioural and physiological assessments in this study were performed using healthy adult mice. However, there may be significant differences in the neural responses to external stimuli between healthy and diseased brains. Future research using disease mouse models is necessary to evaluate whether THz stimulation can effectively alleviate pathological cognitive decline and to further optimize treatment parameters for clinical applications.

In summary, our research provides a THz parameter for improving visual recognition memory. The PRH cortex, located in the superficial brain area, could be used as an appropriate regulatory area for THz wave stimulation. Moreover, we found that THz waves could increase neuronal activity, excitability, functional synaptic plasticity and synaptic transmission. Glutamatergic neurons are the main targets of THz wave exposure, and the accelerated process of neurotransmitter release and vesicle trafficking might be the main contributors to the neural enhancement effect of THz waves. On the one hand, the increase in neuronal firing rates and excitability, along with increased numbers of c-Fos^+^ cells, reflects an overall increase in neural network activation. On the other hand, at the synaptic level, the marked increase in the frequency of both mEPSCs and sEPSCs, together with the increased expression of glutamate receptor subunits and vesicle cycling-related molecules, underscores the potentiation of excitatory synaptic transmission. These changes promoted excitatory networks in the PRH cortex, which might ultimately induce improvements in recognition memory. Future studies are needed to further clarify the modulatory effects of THz waves on the central nervous system and offer new perspectives for their applications.

## Supplementary Information

Supplementary Material 1.

Supplementary Material 2.

## Data Availability

No datasets were generated or analysed during the current study.

## References

[1] Zhang J, Li S, Le W. Advances of terahertz technology in neuroscience: Current status and a future perspective. iScience. 2021;24(12):103548. 10.1016/j.isci.2021.103548 PMC8683584 34977497

[2] Zhao X, Zhang M, Liu Y, Liu H, Ren K, Xue Q, Zhang H, Zhi N, Wang W, Wu S. Terahertz exposure enhances neuronal synaptic transmission and oligodendrocyte differentiation in vitro. iScience. 2021;24(12):103485. 10.1016/j.isci.2021.103485 PMC8649796 34927027

[3] Ma S, Li Z, Gong S, Lu C, Li X, Li Y. High Frequency Electromagnetic Radiation Stimulates Neuronal Growth and Hippocampal Synaptic Transmission. Brain Sci. 2023;13:4. 10.3390/brainsci13040686 PMC10136800 37190651

[4] Liu X, Qiao Z, Chai Y, Zhu Z, Wu K, Ji W, Li D, Xiao Y, Mao L, Chang C, Wen Q, Song B, Shu Y. Nonthermal and reversible control of neuronal signaling and behavior by midinfrared stimulation. Proc Natl Acad Sci USA. 2021;118(10):e2015685118. 10.1073/pnas.2015685118 PMC7958416 33649213

[5] Tan X, Zhong Y, Li R, Chang C. Neuromodulation of Chemical Synaptic Transmission Driven by THz Photons. Research (Washington, D.C.) 2022;2022:0010 10.34133/research.0010 PMC11404318 39285946

[6] Zhang J, He Y, Liang S, Liao X, Li T, Qiao Z, Chang C, Jia H, Chen X. Non-invasive, opsin-free mid-infrared modulation activates cortical neurons and accelerates associative learning. Nat Commun. 2021;12(1):2730. 10.1038/s41467-021-23025-y PMC8115038 33980868

[7] Chao OY, Nikolaus S, Yang YM, Huston JP. Neuronal circuitry for recognition memory of object and place in rodent models. Neurosci Biobehav Rev. 2022;141:104855. 10.1016/j.neubiorev.2022.104855 PMC10542956 36089106

[8] Burwell RD. Borders and cytoarchitecture of the perirhinal and postrhinal cortices in the rat. J Comp Neurol. 2001;437(1):17–41. 10.1002/cne.1267 11477594

[9] Banks PJ, Warburton EC, Brown MW, Bashir ZI. Mechanisms of synaptic plasticity and recognition memory in the perirhinal cortex. Prog Mol Biol Transl Sci. 2014;122:193–209. 10.1016/B978-0-12-420170-5.00007-6 24484702

[10] Tan SZ, Tan PC, Luo LQ, Chi YL, Yang ZL, Zhao XL, Zhao L, Dong J, Zhang J, Yao BW, Xu XP, Tian G, Chen JK, Wang H, Peng RY. Exposure Effects of Terahertz Waves on Primary Neurons and Neuron-like Cells Under Nonthermal Conditions. Biomed Environ Sci: BES. 2019;32(10):739–54. 10.3967/bes2019.094 31843044

[11] Song L, He Z, Dong J, Wang H, Zhang J, Yao B, Xu X, Wang H, Zhao L, Peng R. THz Waves Improve Spatial Working Memory by Increasing the Activity of Glutamatergic Neurons in Mice. Cells. 2025;14(5):370. 10.3390/cells14050370 PMC11898596 40072098

[12] Zhao L, Hao Y-H, Peng R-Y. Advances in the biological effects of terahertz wave radiation. Mil Med Res. 2014;1(1):26. 10.1186/s40779-014-0026-x PMC4340277 25722878

[13] Cherkasova OP, Serdyukov DS, Nemova EF, Ratushnyak AS, Kucheryavenko AS, Dolganova IN, Xu G, Skorobogatiy M, Reshetov IV, Timashev PS, Spektor IE, Zaytsev KI, Tuchin VV. Cellular effects of terahertz waves. J Biomed Opt. 2021;26(9):090902. 10.1117/1.JBO.26.9.090902 PMC8483303 34595886

[14] Alexandrov BS, Rasmussen KO, Bishop AR, Usheva A, Alexandrov LB, Chong S, Dagon Y, Booshehri LG, Mielke CH, Phipps ML, Martinez JS, Chen HT, Rodriguez G. Non-thermal effects of terahertz radiation on gene expression in mouse stem cells. Biomed Opt Express. 2011;2(9):2679–89. 10.1364/BOE.2.002679 PMC3184876 21991556

[15] Alexandrov BS, Phipps ML, Alexandrov LB, Booshehri LG, Erat A, Zabolotny J, Mielke CH, Chen HT, Rodriguez G, Rasmussen KO, Martinez JS, Bishop AR, Usheva A. Specificity and heterogeneity of terahertz radiation effect on gene expression in mouse mesenchymal stem cells. Sci Rep. 2013;3:1184. 10.1038/srep01184 PMC3560359 23378916

[16] Steinzeig A, Molotkov D, Castren E. Chronic imaging through transparent skull in mice. PLoS ONE. 2017;12(8):e0181788. 10.1371/journal.pone.0181788 PMC5559068 28813435

[17] Yuan Z, Qi Z, Wang R, Cui Y, An S, Wu G, Feng Q, Lin R, Dai R, Li A, Gong H, Luo Q, Fu L, Luo M. A corticoamygdalar pathway controls reward devaluation and depression using dynamic inhibition code. Neuron. 2023;111(23):3837–e38535. 10.1016/j.neuron.2023.08.022 37734380

[18] Al-Absi AR, Thambiappa SK, Khan AR, Glerup S, Sanchez C, Landau AM, Nyengaard JR. Df(h22q11)/+ mouse model exhibits reduced binding levels of GABA(A) receptors and structural and functional dysregulation in the inhibitory and excitatory networks of hippocampus. Mol Cell Neurosci. 2022;122:103769. 10.1016/j.mcn.2022.103769 35988854

[19] Dong Z, Chen W, Chen C, Wang H, Cui W, Tan Z, Robinson H, Gao N, Luo B, Zhang L, Zhao K, Xiong WC, Mei L. CUL3 Deficiency Causes Social Deficits and Anxiety-like Behaviors by Impairing Excitation-Inhibition Balance through the Promotion of Cap-Dependent Translation. Neuron. 2020;105(3):475–90. e6. 10.1016/j.neuron.2019.10.035 PMC7007399 31780330

[20] Gao Y, Gao D, Zhang H, Zheng D, Du J, Yuan C, Mingxi M, Yin Y, Wang J, Zhang X, Wang Y. BLA DBS improves anxiety and fear by correcting weakened synaptic transmission from BLA to adBNST and CeL in a mouse model of foot shock. Cell Rep. 2024;43(2):113766. 10.1016/j.celrep.2024.113766 38349792

[21] K BD. Long-term potentiation in the in vitro perirhinal cortex displays associative properties. Brain Res. 1996;733(2):297–300. 10.1016/0006-8993(96)00789-5 8891314

[22] Ma Z, Wei L, Du X, Hou S, Chen F, Jiao Q, Liu A, Liu S, Wang J, Shen H. Two-photon calcium imaging of neuronal and astrocytic responses: the influence of electrical stimulus parameters and calcium signaling mechanisms. J Neural Eng. 2021;18(4):046096. 10.1088/1741-2552/ac0b50 34130271

[23] Aino Sormunen EK, Alitalo K, Saksela K. Nihay Laham-Karam, Seppo Ylä-Herttuala, Comparison of Automated and Traditional Western Blotting Methods. Methods protocols. 2023;6:43. 10.3390/mps6020043 PMC10142486 37104025

[24] Barker GR, Warburton EC. NMDA receptor plasticity in the perirhinal and prefrontal cortices is crucial for the acquisition of long-term object-in-place associative memory. J Neurosci. 2008;28(11):2837–44. 10.1523/JNEUROSCI.4447-07.2008 PMC6670687 18337414

[25] Hayashi MK. Structure-Function Relationship of Transporters in the Glutamate-Glutamine Cycle of the Central Nervous System. Int J Mol Sci. 2018;19(4):1177. 10.3390/ijms19041177 PMC5979278 29649168

[26] Rizo J. Molecular Mechanisms Underlying Neurotransmitter Release. Annu Rev Biophys. 2022;51:377–408. 10.1146/annurev-biophys-111821-104732 PMC9490555 35167762

[27] Wang C, Zhang K, Cai B, Haller JE, Carnazza KE, Hu J, Zhao C, Tian Z, Hu X, Hall D, Qiang J, Hou S, Liu Z, Gu J, Zhang Y, Seroogy KB, Burre J, Fang Y, Liu C, Brunger AT, Li D, Diao J. VAMP2 chaperones alpha-synuclein in synaptic vesicle co-condensates. Nat Cell Biol. 2024;26(8):1287–95. 10.1038/s41556-024-01456-1 PMC11786254 38951706

[28] Wang W, Wang F, Liu J, Zhao W, Zhao Q, He M, Qian BJ, Xu Y, Liu R, Liu SJ, Liu W, Liu J, Zhou XF, Wang TH. SNAP25 ameliorates sensory deficit in rats with spinal cord transection. Mol Neurobiol. 2014;50(2):290–304. 10.1007/s12035-014-8642-8 24519330

[29] Cousin MA. Synaptophysin-dependent synaptobrevin-2 trafficking at the presynapse-Mechanism and function. J Neurochem. 2021;159(1):78–89. 10.1111/jnc.15499 34468992

[30] Brunger AT, Choi UB, Lai Y, Leitz J, Zhou Q. Molecular Mechanisms of Fast Neurotransmitter Release. Annu Rev Biophys. 2018;47:469–97. 10.1146/annurev-biophys-070816-034117 PMC6378885 29792815

[31] Liu M, Liu J, Liang W, Lu B, Fan P, Song Y, Wang M, Wu Y, Cai X. Recent advances and research progress on microsystems and bioeffects of terahertz neuromodulation. Microsyst Nanoeng. 2023;9:143. 10.1038/s41378-023-00612-1 PMC10643571 38025884

[32] Kirichuk VF, Efimova NV, Andronov EV. Effect of high power terahertz irradiation on platelet aggregation and behavioral reactions of albino rats. Bull Exp Biol Med. 2009;148(5):746–9. 10.1007/s10517-010-0807-5 20396783

[33] Kirichuk VF, Antipova ON, Krylova YA. Effect of continuous irradiation with terahertz electromagnetic waves of the NO frequency range on behavioral reactions of male albino rats under stress conditions. Bull Exp Biol Med. 2014;157(2):184–9. 10.1007/s10517-014-2521-1 24952484

[34] Bondar NP, Kovalenko IL, Avgustinovich DF, Khamoyan AG, Kudryavtseva NN. Behavioral effect of terahertz waves in male mice. Bull Exp Biol Med. 2008;145(4):401–5. 10.1007/s10517-008-0102-x 19110578

[35] Qi M, Liu R, Li B, Wang S, Fan R, Zhao X, Xu D. Behavioral Effect of Terahertz Waves in C57BL/6 Mice. Biosensors (Basel). 2022;12(2):79. 10.3390/bios12020079 PMC8869163 35200340

[36] Wang M, Cao J, Amakye WK, Gong C, Li Q, Ren J. Mid infrared light treatment attenuates cognitive decline and alters the gut microbiota community in APP/PS1 mouse model. Biochem Biophys Res Commun. 2020;523(1):60–5. 10.1016/j.bbrc.2019.12.015 31831179

[37] Magee JC, Grienberger C. Synaptic Plasticity Forms and Functions. Annu Rev Neurosci. 2020;43:95–117. 10.1146/annurev-neuro-090919-022842 32075520

[38] Miranda M, Bekinschtein P. Plasticity Mechanisms of Memory Consolidation and Reconsolidation in the Perirhinal Cortex. Neuroscience. 2018;370:46–61. 10.1016/j.neuroscience.2017.06.002 28603027

[39] Hoffman GE, Lee WS, Smith MS, Abbud R, Roberts MM, Robinson AG, Verbalis JG. c-Fos and Fos-related antigens as markers for neuronal activity: perspectives from neuroendocrine systems. NIDA Res Monogr. 1993;125:117–33. 8341361

[40] Koyama S, Nakayama K, Mochizuki A, Dantsuji M, Nakamura S, Maki K, Inoue T. Optical imaging of neurons related to fictive swallowing using GCaMP6f in an arterially perfused rat preparation. J Oral Biosci. 2023;65(1):126–31. 10.1016/j.job.2023.01.009 36738967

[41] Kaszas A, Szalay G, Slezia A, Bojdan A, Vanzetta I, Hangya B, Rozsa B, O’Connor R, Moreau D. Two-photon GCaMP6f imaging of infrared neural stimulation evoked calcium signals in mouse cortical neurons in vivo. Sci Rep. 2021;11(1):9775. 10.1038/s41598-021-89163-x PMC8105372 33963220

[42] Sun Y, Geng J, Fan Y, Li Y, Zhong Y, Cai J, Liu X, Wang S, Gong Y, Chang C, Yang Y, Fan C. A Non-Invasive and DNA-free Approach to Upregulate Mammalian Voltage-Gated Calcium Channels and Neuronal Calcium Signaling via Terahertz Stimulation. Adv Sci (Weinh). 2024;11(47):e2405436. 10.1002/advs.202405436 PMC11653603 39435751

[43] Zhao L, Yi R, Liu S, Chi Y, Tan S, Dong J, Wang H, Zhang J, Wang H, Xu X, Yao B, Wang B, Peng R. Biological responses to terahertz radiation with different power density in primary hippocampal neurons. PLoS ONE. 2023;18(1):e0267064. 10.1371/journal.pone.0267064 PMC9858065 36662735

[44] Wilmink GJ, Rivest BD, Roth CC, Ibey BL, Payne JA, Cundin LX, Grundt JE, Peralta X, Mixon DG, Roach WP. In vitro investigation of the biological effects associated with human dermal fibroblasts exposed to 2.52 THz radiation. Lasers Surg Med. 2011;43(2):152–63. 10.1002/lsm.20960 20740621

[45] Kelleher RJ, Govindarajan A, Tonegawa S. Translational regulatory mechanisms in persistent forms of synaptic plasticity. Neuron. 2004;44(1):59–73. 10.1016/j.neuron.2004.09.013 15450160

[46] Lisman J, Yasuda R, Raghavachari S. Mechanisms of CaMKII action in long-term potentiation. Nat Rev Neurosci. 2012;13(3):169–82. 10.1038/nrn3192 PMC4050655 22334212

