# Supplementary material for: Terahertz wave stimulation improves visual recognition memory via the activation of excitatory neurons in the perirhinal cortex
Source: Cell Commun Signal. 2026 Jul 6;24:517. doi: 10.1186/s12964-026-03051-3 (PMC13628827; doi:10.1186/s12964-026-03051-3)
Supplement: Supplementary file 1 [file 12964_2026_3051_MOESM2_ESM.docx]

Terahertz Waves Stimulation Improves Visual Recognition Memory via Activation of Excitatory Neurons in Perirhinal Cortex

Hui Wang^a,1^, Zhiwei He^a,1^, Lequan Song^b,1^, Ji Dong^a^, Junmiao Pan^a^, Wenjing Cheng^a^, Rui Wang^a^, Yuqing Wang^a^，Li Zhao^a,^*, Ruiyun Peng^a,^*

*^a^ Beijing Institute of Radiation Medicine, 100850, China*

*^b^ Bioinformatics Center of AMMS, Beijing 100850, China*

** Corresponding author.*

*E-mail address: lillyliz@163.com(L.Zhao.);* *ruiyunpeng18@126.com(R.Peng.).*

^1^ *These authors contributed equally to this work.*

**Fig. S1.** Mice irradiated with THz waves. (a) Mouse skull after transparency. (b) Design of mouse fixation device. (c) In vivo THz wave exposure in mice via optical fiber.

**Fig. S2.** THz exposure of brain slices. (a) Schematic diagram of brain slices irradiated with THz waves. (b) Photograph of brain slices irradiated with THz waves. (c) Timeline of in vitro experiments.

**Fig. S3.** Temperature changes in brain slice during 60 min of THz waves exposure
